# Early Triassic marine reptile representing the oldest record of unusually small eyes in reptiles indicating non-visual prey detection

**DOI:** 10.1038/s41598-018-37754-6

**Published:** 2019-01-24

**Authors:** Long Cheng, Ryosuke Motani, Da-yong Jiang, Chun-bo Yan, Andrea Tintori, Olivier Rieppel

**Affiliations:** 1 Wuhan Centre of China Geological Survey, Wuhan, Hubei 430023 P. R. China; 2 Department of Earth and Planetary Sciences, University of California, Davis, CA 95616 USA; 3 Laboratory of Orogenic Belt and Crustal Evolution, MOE, Department of Geology and Geological Museum, Peking University, Yiheyuan Str. 5, Beijing, 100871 P. R. China; 4 Dipartimento di Scienze della Terra, Università degli Studi di Milano, Via Mangiagalli, 34-20133 Milano, Italy; 5 Center of Integrative Research, The Field Museum, Chicago, IL 60605-2496 USA

## Abstract

The end-Permian mass extinction (EPME) led to reorganization of marine predatory communities, through introduction of air-breathing top predators, such as marine reptiles. We report two new specimens of one such marine reptile, *Eretmorhipis carrolldongi*, from the Lower Triassic of Hubei, China, revealing superficial convergence with the modern duckbilled platypus (*Ornithorhynchus anatinus*), a monotreme mammal. Apparent similarities include exceptionally small eyes relative to the body, snout ending with crura with a large internasal space, housing a bone reminiscent of os paradoxum, a mysterious bone of platypus, and external grooves along the crura. The specimens also have a rigid body with triangular bony blades protruding from the back. The small eyes likely played reduced roles during foraging in this animal, as with extant amniotes (group containing mammals and reptiles) with similarly small eyes. Mechanoreceptors on the bill of the animal were probably used for prey detection instead. The specimens represent the oldest record of amniotes with extremely reduced visual capacity, utilizing non-visual cues for prey detection. The discovery reveals that the ecological diversity of marine predators was already high in the late Early Triassic, and challenges the traditional view that the ecological diversification of marine reptiles was delayed following the EPME.

## Introduction

The modern marine ecosystem would be incomplete without air-breathing, tetrapod predators, such as cetaceans and pinnipeds^1^, which dominate the list of the heaviest marine predators. The air-breathing predators, or marine tetrapod vertebrates, first emerged following the end-Permian mass extinction that occurred about 252 million years ago (ma) and revolutionized the composition of marine predatory communities^2–5^. It has been thought that marine tetrapods gradually increased their diversity toward the middle of the Middle Triassic (about 240 ma), mirroring the supposedly slow tempo of “delayed recovery”^6–8^, but emerging evidence suggests that they diversified faster both taxonomically^4^ and ecologically^5^. Most of the earliest marine reptiles were Ichthyosauromorpha^4^, a clade that eventually gave rise to fish-shaped ichthyosaurs by the latest Middle Triassic^9^. In its early history, Ichthyosauromorpha comprised a clade of peculiar marine reptiles called Hupehsuchia, the sister taxon of the main clade Ichthyosauriformes^10^.

Hupehsuchia has been known since 1959^11^ but remained poorly understood until the recent rush of discoveries made by the Wuhan Centre of China Geological Survey (WGSC)^5,12–15^ (see also^16^). Hupehsuchia are now noted for their high taxonomic diversity despite their narrow temporal and geographical ranges^15^. The clade was endemic to a large lagoon located near the northern edge of the Yangtze Carbonate Platform, which spanned more than 1200 km east to west and 500 km north to south^17^. The fossil lagoon is now placed in the central-west region of Hubei Province, China, being split between Nanzhang County in Xiangyang City, and Yuan’an County in Yichang City. Hupehsuchian fossils are almost exclusively found in the top 20 m of the Third Member of the Jialingjiang Formation (latest Spathian, Early Triassic), immediately below the Lower/Middle Triassic boundary (247.2 ma). Five monotypic genera have so far been recognized in this clade^15^, and some specimens likely representing additional taxa are under study. The lagoon was also inhabited by at least two sauropterygian marine reptiles, *Hanosaurus hupehensis*^18^ and ‘*Keichousaurus*’ *yuananensis*^19^, and one ichthyosauriform, *Chaohusaurus zhangjiawanensis*^20^.

**Figure 1:**
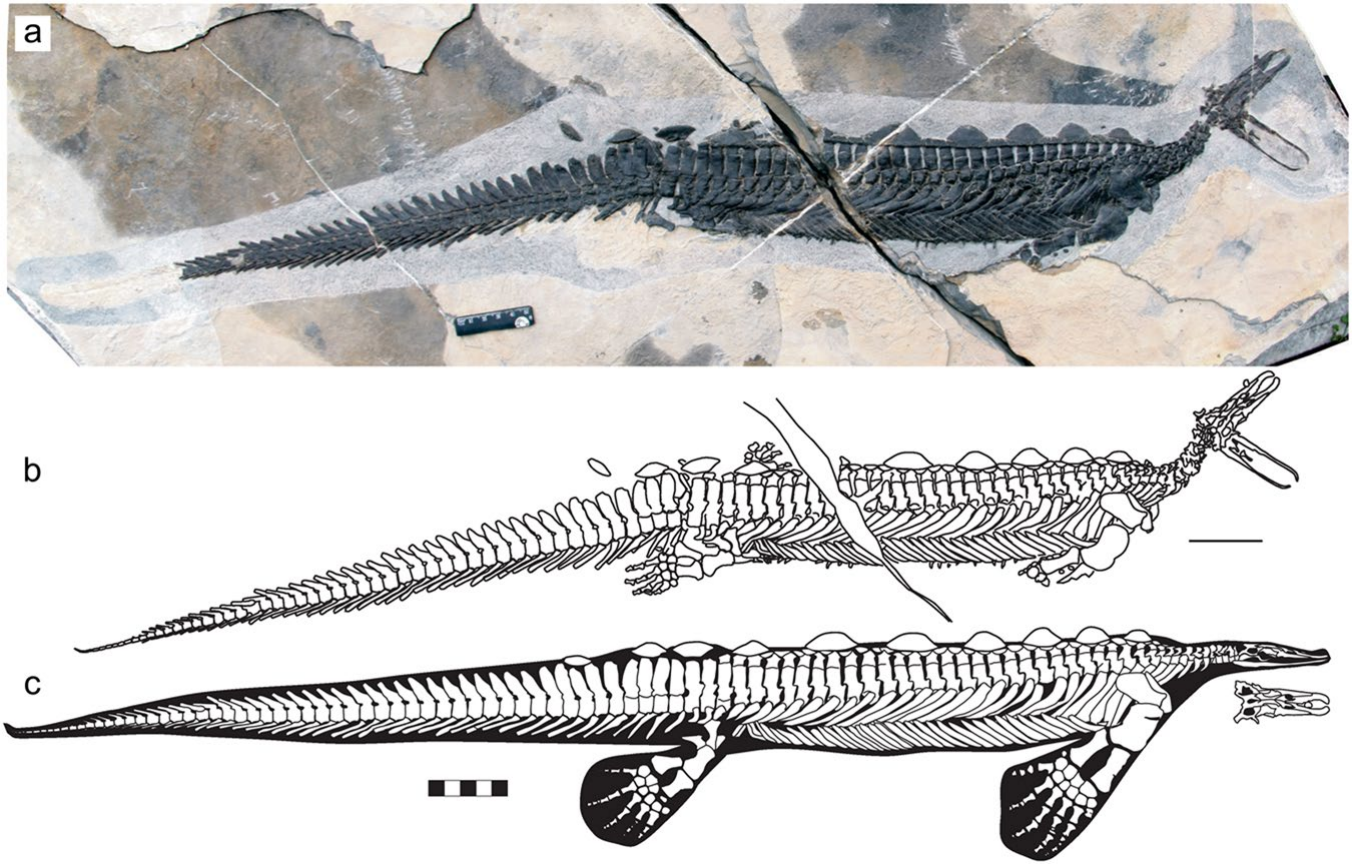
First nearly complete specimen of the rare hupehsuchian *Eretmorhipis carrolldongi* (YAGM V 1401), revealing an unusually small skull. (**a**) photograph. (**b**) outlines of the bones and impressions. (**c**) skeletal reconstruction, with flippers from the holotype. The ruler is 5 cm long.

**Figure 2:**
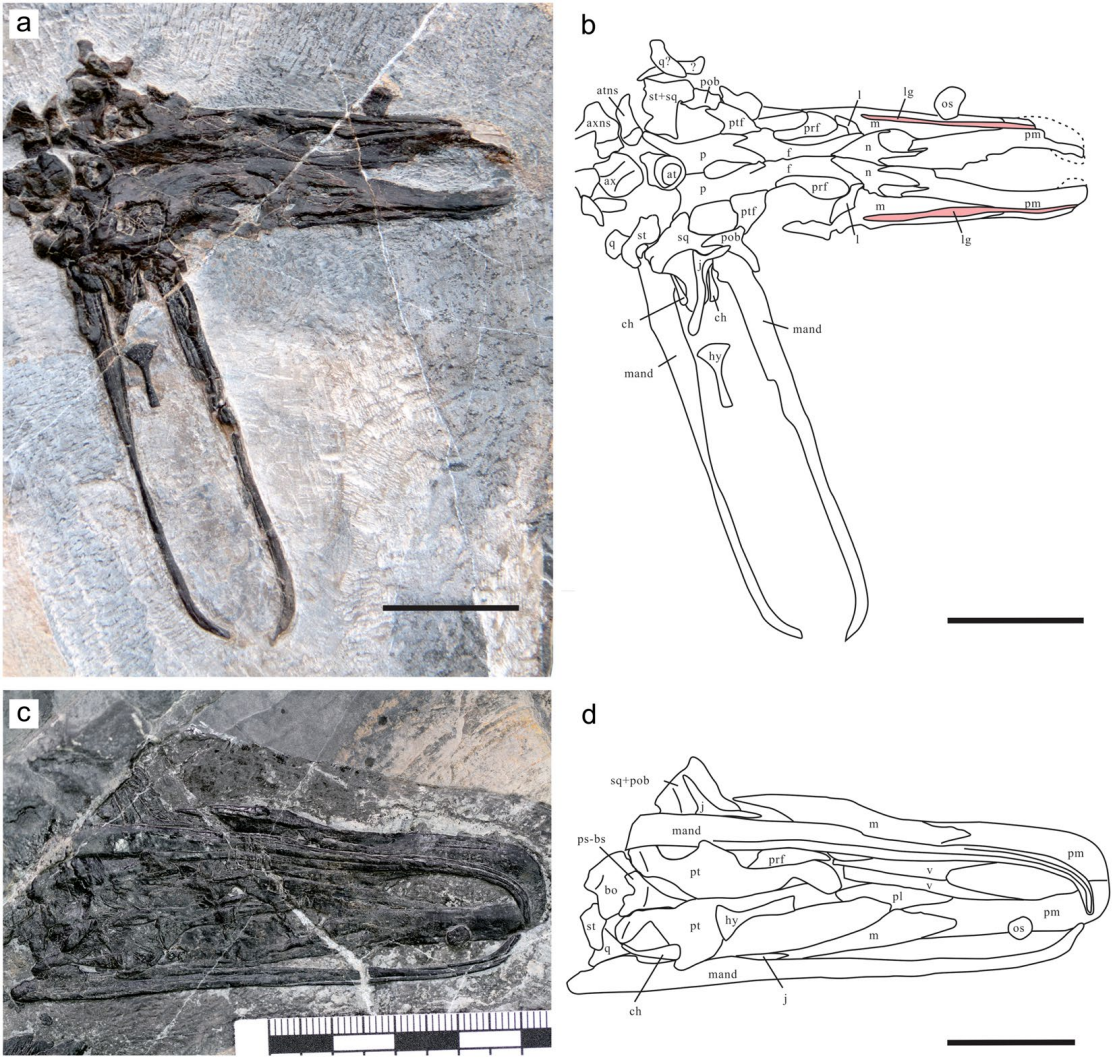
The skull and mandible of *Eretmorhipis carrolldongi* in two new specimens. (**a**) and (**b**) YAGM V 1401, in dorsal view. (**c**) and (**d**) WGSC V 1601, in ventral view. Scale bars are 20 mm long. Symbols: at, atlas; atns, atlantal neural spine; ax, axis; axnp, axial neural spine; bh, basihyal lingual process; ch, ceratohyal; f, frontal; j, jugal; l, lacrimal; lg, labial groove for labial cartilage; m, maxilla; mand, mandibular rami; n, nasal; os, bone resembling os paradoxum; p, parietal; palatal, unidentified palatal bones; pl, palatine; pm, premaxilla; pob, postorbital; prf, prefrontal; ps-bs, parasphenoid-basisphenoid complex; pt, pterygoid; ptf, postfrontal; q, quadrate; sq, squamosal; st, supratemporal; v, vomer.

WGSC collected two new hupehsuchian specimens that reveal surprising cranial morphology from the dorsal and ventral aspects, respectively. They are referred to *Eretmorhipis carrolldongi*, for which only headless specimens have been known^15^. The holotype is a nearly complete skeleton lacking the neck and cranium, exposed from the left-dorsal aspect, while the only other specimen to date was made of postcranial bone impressions. One of the new specimens is a nearly complete skeleton only lacking parts of the limbs (YAGM V 1401, Yuan’an Geological Museum), excavated in a quarry in Hekou, Yuan’an County that has become a field paleontological display (Figs 1 and 2). The other specimen, from about 3 km northwest of the locality above, only preserves the anterior part of the body (WGSC V 1601) (Fig. 2). The specific referral is based on diagnostic features described in Methods. The skull of *E. carrolldongi* shares a suite of strange morphological resemblance to that of the duckbilled platypus (*Ornithorhynchus anatinus*), an extant monotreme mammal in Australia, in the overall construction of the snout and the smallness of the eye. The purpose of the paper is to assess the anatomical features of *E. carrolldongi* that are indicative of its ecology, including the superficial resemblance to *O. anatius*, in order to provide further information on the ecological diversification of early marine reptiles after EPME.

## Results

### Orbit proportion

**Figure 3:**
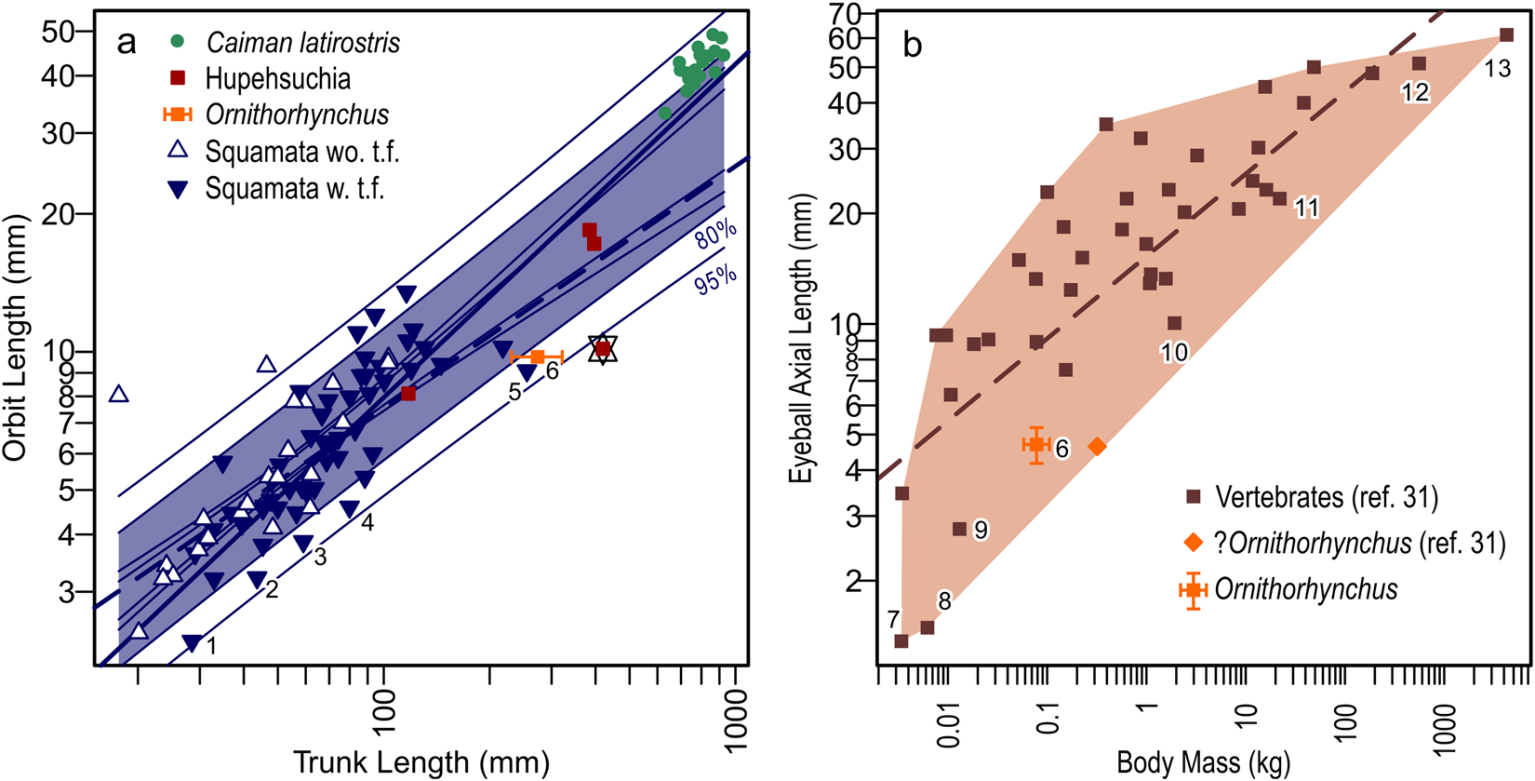
Comparison of relative eye size. (**a**) Orbit versus body trunk lengths in hupehsuchians, *Ornithorhynchus*, squamates, and a semiaquatic archosaur (*Caiman latirostris*). (**b**) Eyeball axial length versus body mass in extant vertebrates. Symbols and colors: blue filled triangles, Squamata with tongue-flicking; blue open triangles, Squamata without tongue-flicking; dark brown, vertebrates; green circles *Caiman latirostris*; orange diamond and square, *Ornithorhynchus*; red-brown square, Hupehsuchia. *Eretmorhipis* has been annotated with double-triangles (or David’s star). Thick solid line represents phylogenetically informed Standardized Major Axis regression for squamates, and thick dotted lines phylogenetically informed Generalized Least Square regression for the same. Thin lines represent 95% and 80% confidence and prediction intervals from the Ordinary Least Square regression. Light-brown area in (**b**) represents the convex hull surrounding the vertebrate data. Published data were used for squamates^40^ and *C. latirostris*^39^. The presence/absence of tongue-flicking in squamates follows ref.^50^. For *Ornithorhynchus* in (**a**), orbit and head size data^51^ were plotted against the median and 95% quantile bar for trunk length of the species (n = 256)^49^. See Methods for the data in (**b**). The *Ornithorhynchus* data point from ref.^23^ seems to mix the eye size of a juvenile and average body mass of adults, and therefore dislocated to the right. See Table 1 for the identities of species numbered 1–13.

*Eretmorhipis* had very small eyes. Measurements reveal that the size of the orbit relative to the trunk was unusually small in *Eretmorhipis*, only rivaled by those squamates with the smallest eyes for the body outside of snakes and similarly elongated forms (Fig. 3a). As seen in the plot, *Eretmorhipis* lies outside of the 95% prediction interval for squamates. In contrast, other hupehsuchians had a relative eye sizes that were within the range for typical squamates (Fig. 3a). *Eretmorhipis* and *Hupehsuchus* have similar body sizes but the orbit is twice as large in the latter genus.

### Snout morphology

**Figure 4:**
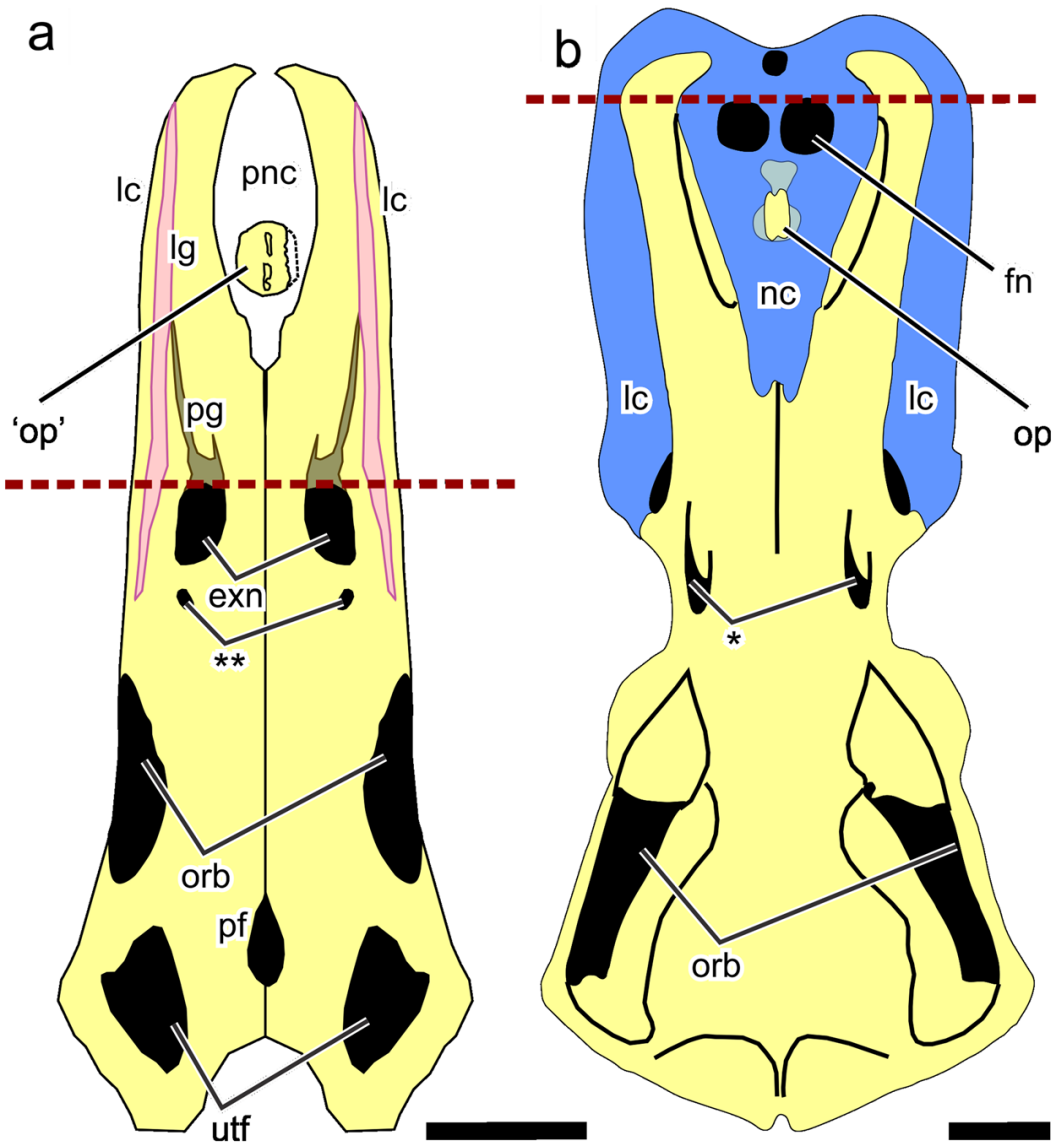
Dorsal view of the skulls of *Ornithorhynchus anatinus* and *Eretmorhipis carrolldongi*. (**a**) *E. carrolldongi*. (**b**) *O. anatinus*, based on a published figure. Colors: black, cranial fenestrations; brown, prenasal groove; light blue, prenasal and labial cartilages; light yellow, bone; pink, groove for labial cartilage. Symbols: exn^23^, external naris; fn, fenestra naria, located below exn; lc, labial cartilage; lg, labial groove for labial cartilage; nc, nasal capsule; op, os paradoxum; orb, orbit; pf, pineal foramen; pg, prenasal groove; pnc, prenasal cartilage of unknown homology; utf, upper temporal fenestra; *unnamed foramen for passage of a branch of the ethmoidal nerve; **foramen analogous to *. Brown dotted lines mark the anterior extent of the external naris, and therefore of the nasal capsule and associated structures, illuminating that the intercrural space of *Eretmorhipis* is in front of the nasal capsule, unlike in *Ornithorhynchus*. Scale bars are 1 cm long.

**Figure 5:**
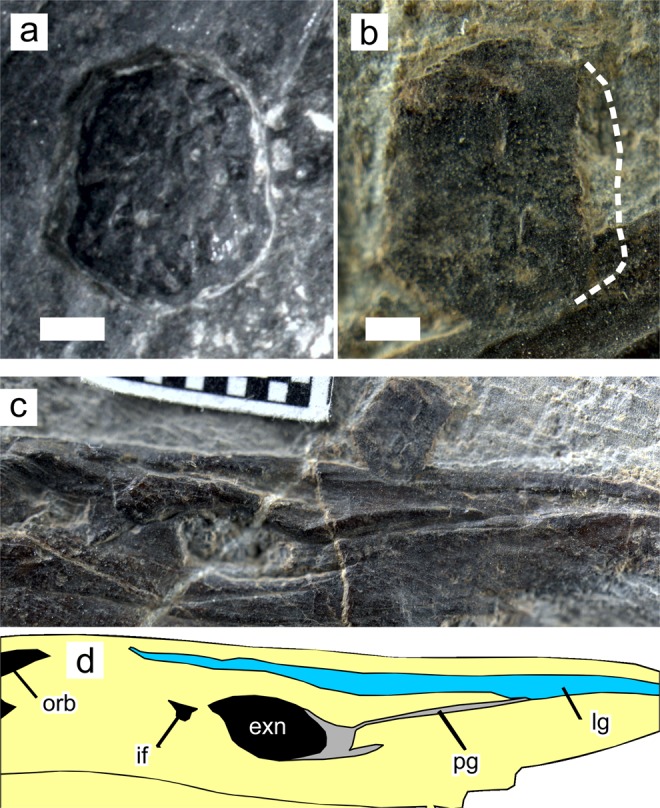
Preorbito-external-narial region of *Eretmorhipis carrolldongi* and a bone resembling os paradoxum. (**a**) ‘Os paradoxum’ of WGSC V 1601, in ventral view with unfinished surface. (**b**) Same of YAGM V 1401, in dorsal view revealing a median ridge; (**c**)-(**d**) Preorbito-external-narial region of YAGM V 1401. Scales for (**a**) and (**b**) are 1 mm, and each square in (**c**) has a side length of 1 mm. See Fig. 4 for symbols.

The snout of *Eretmorhipis* exhibits some gross structural similarities with that of *Ornithorhynchus*. The snout skeleton of *Eretmorhipis* is divided into right and left crura that surround a median oval space (Figs 2–4). This intercrural space houses an isolated bone, whose unfinished surface texture suggests that it was enclosed in cartilage at least ventrally and probably peripherally (Fig. 5a,b). There is a slight ridge on the dorsal surface of this bone, suggesting that it may have supported the bottom of a paired structure (Fig. 5a,b)—but see below for further discussion. A conspicuous groove runs along the labial margin of the maxilla and premaxilla (Fig. 5c,d), resembling a groove that holds the labial cartilage in *Ornithorhynchus*. There is a foramen in front of the orbit (Fig. 5c,d). The foramen is present on both sides of the skull, although the left one has been obscured through a slight posterior displacement of the left maxilla. Such a foramen is unknown in other hupehsuchians, or in most reptiles^21^. Two of the laterally-exposed skulls of *Hupehsuchus* have a hole surrounded by the maxilla, prefrontal, and nasal that probably represents breakage during lateral compression of the skulls. Another laterally exposed specimen (WGSC V 26004) and a dorsally exposed skull (WGSC V 26007) of *Hupehsuchus* lack such an opening.

The mandible of *Eretmorhipis* is unique among hupehsuchians in that the two rami are almost parallel to each other over the posterior ~90% of the mandibular length and then curve rostrally toward the midline, although they do not meet along the midline to form a symphysis (Fig. 2). As a result, the horizontal profile of the mandible matches that of the skull. In other hupehsuchians, the mandibular rami are straight and extremely slender, with a narrow, triangular intermandibular space between them. It was suggested that these rami bowed during lunge feeding, the water force expanding the intermandibular space and pouch therein, as in pelicans^5^. The mandible of *Eretmorhipis* appears to be permanently ossified in a curved shape, and each ramus was slightly thicker than in *Hupehsuchus* to stiffen the structure. There is no sign of cartilage attachments on the mandibular sides.

### Body trunk

The new skeleton (YAGM v1401) reveals the lateral aspect of the trunk of *Eretmorhipis* for the first time, demonstrating that it was a shallow-bodied form unlike the deep-bodied *Hupehsuchus* (Fig. 1). It also shows that the third-layer dermal ossicles protruded dorsally from the body wall, forming triangular blades somewhat reminiscent of the dorsal plates of *Stegosaurus*. There is a total of 10 such triangular blades, giving a zig-zagged appearance to the dorsal outline of the animal (Fig. 1).

The trunk and tail were both almost rigid, leaving only the hip region and the neck for body flexion. In the trunk, thickened ribs and gastralia were closely packed, spanning three to four body segments per element, thus largely limiting flexibility between segments. In the tail, the hemal spines are almost horizontally oriented, again spanning about three body segments each, with little open space between them. The rigidity of the two regions is also indicated by the preservation of fossils: there are now three specimens with the trunk and tail preserved in articulation, and both parts are straight in all three, with a slight bending at the hip.

## Discussion

Size is an important characteristic of eyes. The absolute size of the eye is theoretically correlated with acuity, the eye’s ability to resolve images, and the relationship has been empirically confirmed at least in mammals and actinopterygian fishes^22–25^. The eyes of *Eretmorhipis* had significantly lower resolving power compared to those of *Hupehsuchus*, the latter with an orbit about twice as large relative to comparable body size. Very small eyes, either in absolute size or relative to the body, indicate impoverished vision in any given species^22,23^. The traditional view holds that animals with exceptionally small eyes rely on non-visual sensory cues, especially if their behavior includes activity in reduced light^22,23,25^. This is demonstrated by Fig. 3, where all species with the smallest eyes relative to body size, identified by numbers 1–13, have without exception a combination of (1) enhancement of a sense organ other than vision and (2) activity in visually challenging conditions (Table 1). *Eretmorhipis* had a relative eye size equal to or smaller than those of the 13 numbered species in Fig. 3, representing the oldest record of such small eyes in amniotes. The size is far too small for a vertebrate that relies solely on vision; we consequently infer that some senses other than vision were enhanced in *Eretmorhipis*.

**Table 1:** Reduced-light conditions and alternative senses in vertebrates with the smallest eyes for the body.

Species	Diel activity pattern/Behavior in reduced light	Alternative sense	Fig. 3 Symbols
Blarina brevicauda	Crepuscular, nocturnal^52^	Echolocation^53^	7
Dugong dugon	Cathemeral^54^	Tactile hair^55^	11
Glaphyromorphus nigricaudis	Nocturnal^56^	Tongue-flicking^50^	3
Heloderma sp.	Crepuscular, nocturnal^57^	Tongue-flicking^50^	5
Lepidophyma gaigeae	Staying in cracks^58^	Tongue-flicking^50^	2
Loxodonta africana	Cathemeral^59^	Acoustics^60,61^	12
Megaptera novaeangliae	Cathemeral^62^	Acoustics^63^	13
Meles meles	Crepuscular, nocturnal^64^	Olfaction^64^	10
Microtus pennsylvanicus	Arrhythmic^65^	Olfaction^66^	9
*Ornithorhynchus* *anatinus*	Arrhythmic^67^	Electroreception^67^	6
Scincus mitranus	In-sand swimming^68^	Tongue-flicking^50^	4
Sorex palustris	Crepuscular, nocturnal^69^	Olfaction, Vibrissa^70^	8
Xantusia vigilis	Arrhythmic^71^	Tongue-flicking^50^	1

Numbers 1–13 corresponds to Fig. 3. Diel activity patterns involving activities in dim or dark environments are: arrhythmic, active any time of the day without a pattern; cathemeral, both day and night active; crepuscular, dawn and twilight active; nocturnal, night active.

Soft-tissues are not preserved in fossils in question, so it is impossible to explicitly test which non-visual sense of Eretmorhipis carrolldongi may have been enhanced. However, certain possibilities are reasonably eliminated. Hearing is an unlikely candidate because sound localization in water is difficult for animals with small heads, given that sound travels five times faster in water than in air^26,27^. Also, no specialization is known in the ear region of E. carrolldongi. Chemoreception through tongue flicking is also unlikely because the vomeronasal fenestra is lacking in the palate, when its presence is essential for conveying chemical information from the tongue to vomeronasal organs in tongue-flicking squamates^28^. The nose openings do not exhibit any specialization while there is no evidence for the secondary palate, making special enhancement of olfactory sense unlikely. Chemoreception though taste buds is useful in proximal chemical stimuli, such as food particles from oral processing^28^, but is unsuitable for prey and predator detection. This leaves the tactile sense as the most likely candidate among the traditional five senses. The possibility of electroreception in Eretmorhipis cannot be ruled out. A broad array of vertebrates has electroreceptors. The most common, plesiomorphic electroreception is based on ambulatory cells derived from the lateral line system^29^, which was lost in many lineages including amniotes. Within Amniota, new trigeminal-based electroreception evolved at least twice in aquatic mammals: once in monotremes^30^ and again in the Guiana Dolphin^31^. New examples are expected to be discovered^32^—its presence in a dolphin was unknown until 2012. No reptile is known to have electroreception at this point, but aquatic reptiles have been mentioned among the candidates for future discoveries of electroreception^32^. It is therefore not impossible that Eretmorhipis used electroreception along with mechanoreception.

Despite the remarkable set of structural similarities between the skulls of *Eretmorhipis* and the modern duckbilled platypus (*Ornithorhynchus anatius*), structural similarities do not necessarily imply functional convergence. Most notably, the position of the external nares differs between *Eretmorhipis* and *Ornithorhynchus*—they are located behind the intercrural space in the former, and near the anterior end of the space in the latter. Thus, the space is filled by the nasal capsules in *Ornithorhynchus* but, a similar cartilage in Eretmorhipis, if any, could not have been the nasal capsule. Thus, the nervous system in the bill most likely had different arrangements between the two. For the same reason, the isolated bone in the intercrural space of *Eretmorhipis* is unlikely to have served the same role as os paradoxum of *Ornithorhynchus*, which supports the paraseptal cartilage encasing Jacobson’s organ, and the nasal septum^33,34^. The bone in *Eretmorhipis* could not have supported the vomeronasal organ, which is expected to have been located behind the external naris. The foramen in front of the orbit in *Eretmorhipis* is not homologous with that in *Ornithorhynchus*, given that they are located between different bones. The nerve that this foramen transmitted in *Eretmorhipis* is unknown, although given its position it was most likely a branch of the ophthalmic division of the trigeminal nerve. Therefore, it is difficult to strictly infer that *Eretmorhipis* shared its bill function with *Ornithorhynchus* based on the bill osteology alone.

The permanent bowing of the mandible of *Eretmorhipis* removes the need for quick acceleration during prey capture, which is expected in a lunge-feeding *Hupehsuchus* whose intermandibular space requires the water force to expand it. The absence of quick lunging, which would require detection of prey from a distance, conforms well with the feeding style based on tactile sense suggested for *Eretmorhipis*.

The rigidity of the tail probably reduced its effectiveness as a propulsive organ, forcing *Eretmorhipis* to use the limbs to aid propulsion. The limbs and girdles of *Eretmorhipis* were indeed more robustly built than in other hupehsuchians, and paddle size was expanded by digits that are radially spread, again unlike in other hupehsuchians^15^. The fan-shaped limbs were likely used in both propulsion and maneuvering, as implied by the generic name.

Although it is difficult to assess the degree of night-hunting ability in *Eretmorhipis*, the use of the tactile sense in foraging, with a reduced role of vision, would allow it to hunt in lower light conditions than other hupehsuchians. This inference is supported by Table 1, which, as discussed above, complies with the traditional view that species with exceptionally small eyes relative to the body are active in reduced light, while also enhancing sensory systems other than vision. The relative eye size of *Eretmorhipis* is equal to or smaller than that of the species listed in Table 1.

The interpretation that *Eretmorhipis* foraged under low light conditions is also plausible from an ecological point of view. The lagoon where *Eretmorhipis* lived had at least eight coeval species of marine reptiles, five of which were hupehsuchians, leading to the question how such a high diversity could be sustained in a limited geographical area^5^. One of the possible solutions is the temporal division of resources, i.e., more species can coexist in a single area when foraging at different times of the day. The low-light activity in *Eretmorhipis* would allow such a temporal habitat partitioning. Note that we are not necessarily advocating nocturnality in *Eretmorhipis*—low-light activity occurs not only in nocturnal but also in arrhythmic, cathemeral, and crepuscular species.

**Figure 6:**
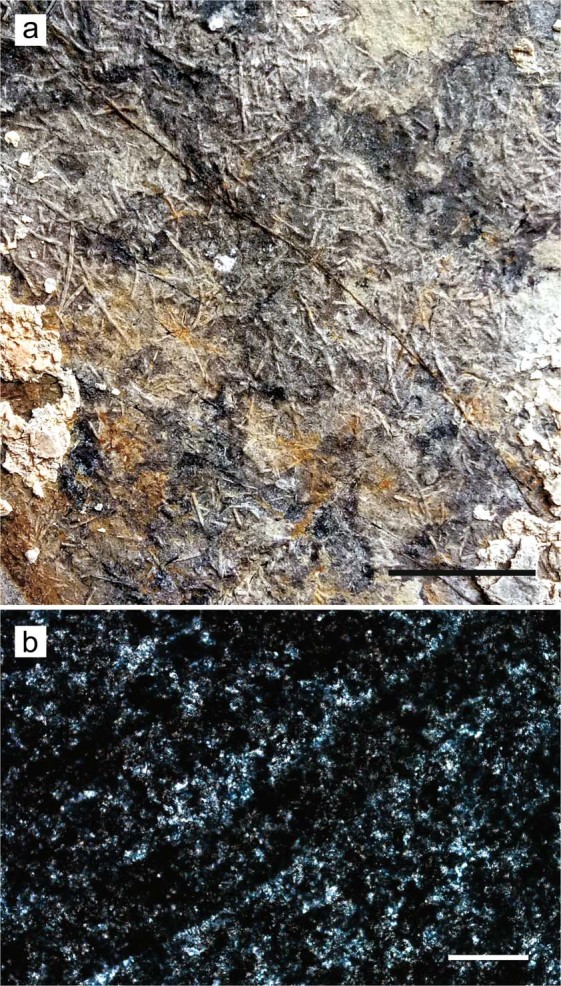
Fecal pellets attributed to shrimps or shrimp-like invertebrates (WGSC MTJ-01). (**a**) Macroscopic view of one of the slabs. (**b**) Cross-sectional view under the microscope. Scale bar is 1 mm in (**a**) and 200 µm in (**b**).

The diet of *Eretmorhipis* is unknown. Non-reptilian fossils are extremely rare in the Nanzhang-Yuan’an Fauna, where not even a single fish scale has been found despite the continued efforts to dissolve matrix in search for microfossils. Recently, fecal pellets have been found in Nanzhang that are best attributed to shrimp-like arthropods (Fig. 6). A cross-section of one such aggregation of fecal pellets did not reveal any structure apart from the outlines of the tubes that are here interpreted as fecal pellets. The size of the fossilized fecal pellets matches those of at least one extant shrimp—it has been reported that the shrimp *Palaemonetes pugio*, with body lengths between 2.2 and 2.5 cm, left fecal pellets that were 50 to 200 µm wide and 1 to 20 mm long^35^, whereas the fossil fecal pellets are about 200 µm wide (Figs. 6b) and 5 mm long (Fig. 6a). The size of the feeding apparatus of *Eretmorhipis* is appropriate for capturing such prey, and shrimps are among the list of prey items of the duckbilled platypus^36^, which hunts in the same way as is here inferred for *Eretmorhipis*. Therefore, shrimps or similar invertebrates are the best candidates for the prey of *Eretmorhipis*.

In sum, *Eretmorhipis* was a slow yet maneuvering swimmer with a rigid body and tail coupled with large fan-shaped propulsive flippers. Its prey probably included shrimp-like arthropods. The reduced role of vision and the likely use of tactile cues allowed the animal to hunt in low-light conditions, thus enabling temporal partitioning of the trophic resources with other marine reptiles.

It has been pointed out that variation in the feeding style of marine reptiles was already very high in the Spathian^5^, the last substage of the Lower Triassic, where six feeding types had been recognized based on the combination of tooth shape, prey capture mode, and feeding sites (pelagic vs. bottom feeder). This was the highest number throughout the Triassic, only rivaled by the early Middle Triassic diversity. Since then, another feeding type, grazer, was recognized in the late Middle Triassic marine reptile *Atopodentatus*^37^, increasing the number of feeding types to six throughout the Early and Middle Triassic.

The new specimens of *Eretmorhipis* suggest that there was yet another feeding ecomorph among the Early Triassic marine reptiles based on the difference in prey detection, i.e., the use of the tactile sense as opposed to the normal visual sense. The essential mode of prey/predator detection in amniotes is to use visual cues, although some species have evolved to augment or replace vision with other senses, as in the 13 species in Table 1. *Eretmorhipis* represents the oldest record of extremely reduced relative eye size in amniotes, as stated earlier. It suggests that amniotes started exploring the use of non-visual cues for prey/predator discrimination at least as early as the Early Triassic. This new feeding strategy renders the number of marine reptile feeding types in the Triassic the highest in the Early Triassic. Although future discoveries may alter these numbers further, it appears safe to state that the number of marine reptile feeding types reached a maximum within the Early Triassic. The traditional view holds that the initial evolution of marine reptiles was slow and gradual until the Middle Triassic^6–8^. The new findings add to the emerging insight that the diversification of marine reptiles was already rapid in the Spathian of the Early Triassic^4,5^.

## Methods

### Taxonomic referral

The specific referral of the two new specimens to *Eretmorhipis carrolldongi* is based on the following six apomorphies: small ulnar flange in olecranon region; pectoral ribs flattened while more posterior ribs thickened; manual and pedal digits radiating, forming fan-shaped paddles; dermal ossicles along the trailing edge of forelimb paddle; third-layer dermal ossicle absent in every second position; third-layer dermal ossicles enlarged, spanning up to four vertebral segments.

### Eye size comparison

It would be ideal to plot the axial length of the eyeball against body mass to compare the relative eye size of animals^23,24^. However, these values are not preserved in fossils so we instead used the orbit length plotted against the snout-vent length to include *Eretmorhipis*. Relative orbit size to the skull length may sometimes be used in comparative visual optics but this metric is not as reliable because it is largely biased by skull morphology. For example, it is not useful in hupehsuchians because their unusually elongated snouts diminish the values below most vertebrates, as seen in the plot in Supplementary Fig. S1 based on published data^38,39]^.

Given that *Eretmorhipis* has a small head relative to the body, one may suspect that the smallness of the head may be biasing the comparison. However, that is very unlikely. The relative head length to trunk length in Hupehsuchia varies across the range known for extant squamates (Supplementary Fig. S1), so Hupehsuchia in general are not small-headed and our comparisons of Hupehsuchia against Squamata is not biased by the relative skull size differences. Note especially that our squamate data contain several squamate species with smaller relative skull sizes than in *Eretmorhipis*. As previous authors have established, the absolute size is the most important characteristic of an eyeball that affects the visual capacity. The head size of vertebrate animals therefore may confine visual capacity by limiting the maximum possible eye size. This limitation, however, is usually irrelevant because most vertebrates do not shrink their heads so far as to sacrifice their visual capacities, unless vision is no longer important. Thus, eye size may be confining the minimal head size in vison-oriented vertebrates, instead of the other way around. Also, substantial shortening of the skull is possible without shirking the eye size, simply by enlarging the proportion of the eyes relative to the skull (e.g., short-beaked birds have small relative head size in comparisons to squamates, but their relative eye sizes are not as small)—another reason why the skull length is a weak proxy for body size.

### Regression analysis

Regression lines given in Fig. 3a were calculated based on published morphological data^40^ and a molecular phylogenetic hypothesis^41^ of squamates. Two methods, phylogenetically informed Reduced Major Axis regression (PRMA) and Phylogenetic Generalized Least Squares (PGLS) were performed. Only those species that are present in both the tree and morphological data were used in the analysis (n = 57). Calculations were done in R^42^, with the packages Ape^43^, and Phytools^44^. Regression in Fig. 3b was performed based on the data explained below and a time-calibrated molecular phylogenetic hypothesis obtained from TimeTree^45^. PGLS and Ordinary Least Square (OLS) were used. Results from PRMA is not reported because of an internal error of the function during computation. Prediction intervals (95%) were calculated using OLS in both plots because GLS is not designed to provide prediction intervals.

### Replot of relative eye size

There was a recent suggestion that the eyes of *Ornithorhynchus* were not small compared to those of other vertebrates of the same body mass^46^ but this is an artifact of using incompatible variables—the authors compared the antero-posterior diameter of the orbit of *Ornithorhynchus* with the eyeball axial lengths in other species, whereas the axial length of the eyeball in *Ornithorhynchus* is less than its diameter^47,48^, which in turn is less than the orbit diameter. We therefore replotted the relative eye size of *Ornithorhynchus* to body mass using the vertebrate data in ref.^23^ and *Ornithorhynchus* data in other studies (Fig. 3b). The data set in ref.^23^ contained body mass data of various quality, ranked from Group 1 to 7 depending on their accuracy. Many of the data points combined an eyeball measurement from one study with a body mass of the same species given in another study. Therefore, there is a large potential for mismatch between the eyeball size and body mass. *Ornithorhynchus* was included in the original data^23^ but this data point was not adopted by ref.^46^, probably for a reason that the body mass (1.46 kg), taken from an encyclopedia, was too large for the eyeball size (4.64 mm), which most likely was derived from a juvenile individual (see below; Fig. 3b). We therefore retained only those data points for which the eyeball axial length and body mass had been derived from a single study (n = 51). We then added a data point for *Ornithorhynchus* based on a paper^47^ that reported an eyeball diameter of 6 mm for a juvenile individual with a total length of 295 mm. The diameter was converted to axial length using the ratio between the two, measured from a cross-section photograph of the eyeball in the same paper, and using another ratio from a cross-section photograph of fresh-frozen eyeball in a different paper^48^. Two different ratios were used because there is a possibility that the exposed part of the eyeball in the first photograph may be somewhat desiccated based on the appearance. This procedure resulted in a range of 4.2 to 5.2 mm for the eyeball axial length—note that this range contains the eyeball axial length given in ref.^23^, suggesting that that value was also taken from a juvenile. The body total length was converted to body mass using a regression between the two in *Ornithorhynchus* based on the data presented in ref.^49^ (n = 256), with 95% prediction interval. This resulted in a body mass of 317 (229–439) g.

## Supplementary information

Supplementary Information
