# Supplementary material for: Early Triassic marine reptile representing the oldest record of unusually small eyes in reptiles indicating non-visual prey detection
Source: Sci Rep. 2019 Jan 24;9:152. doi: 10.1038/s41598-018-37754-6 (PMC6345829; doi:10.1038/s41598-018-37754-6)
Supplement: Supplementary file 1 — Supplementary Information [file 41598_2018_37754_MOESM1_ESM.pdf]

SUPPLEMENTARY INFORMATION for

Early Triassic marine reptile representing the oldest record of unusually small eyes in reptiles indicating non-visual prey detection

Long Cheng[1,*], Ryosuke Motani[2,*], Da-yong Jiang[3], Chun-bo Yan[1], Andrea Tintori[4], Olivier Rieppel[5]

[1].Wuhan Centre of China Geological Survey, Wuhan, Hubei 430023, P. R. China.

[2].Department of Earth and Planetary Sciences, University of California, Davis, CA 95616, U.S.A.

[3].Laboratory of Orogenic Belt and Crustal Evolution, MOE; Department of Geology and Geological Museum, Peking University, Yiheyuan Str. 5, Beijing 100871, P.R. China.

[4]. Dipartimento di Scienze della Terra, Università degli Studi di Milano, Via Mangiagalli 34-20133 Milano, Italy.

[5].Center of Integrative Research, The Field Museum, Chicago, IL 60605-2496, U. S. A.

*Correspondece to rmotani@ucdavis.edu or

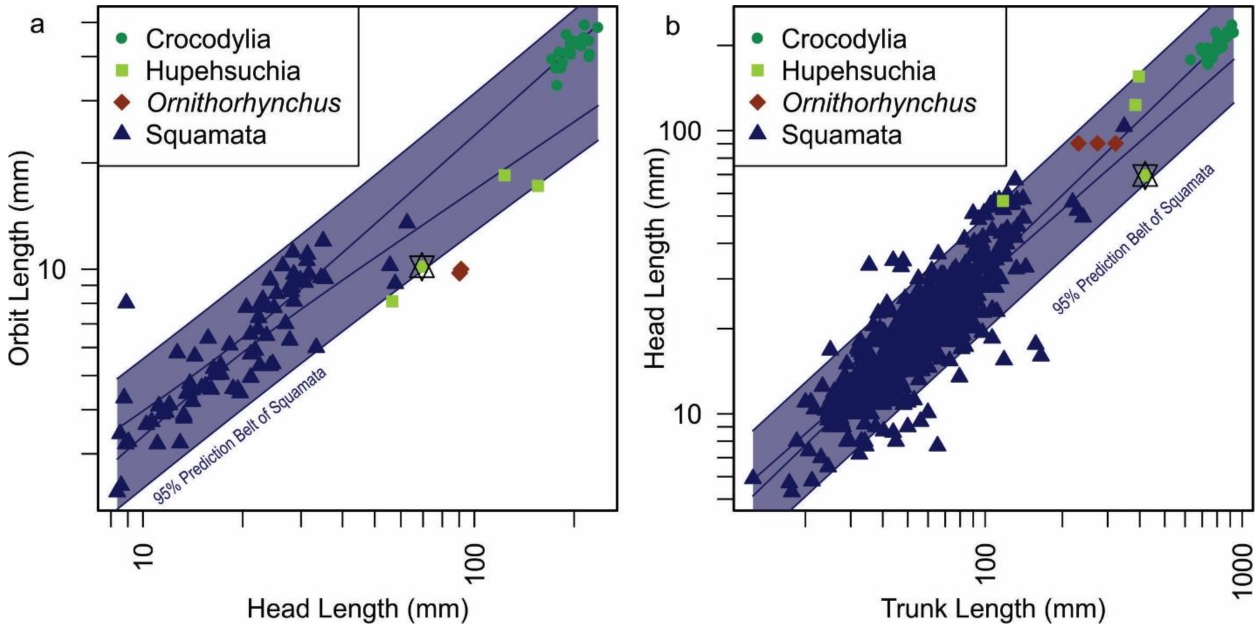

Supplementary Fig. S1. Plots involving the head length in squamates and crocodylians, with hupehsuchians and *Ornithorhynchus* added for comparison. (a) Orbit Length against Head Length. (b) Head Length against Trunk Length. Star of David marks *Eretmorhipis*. See Methods for the data sources.
